# Toward an intravaginal device to detect risk of preterm labor: a user-centered design approach in Sub-Saharan Africa

**DOI:** 10.1186/s12978-022-01478-8

**Published:** 2022-07-30

**Authors:** Emma Smith, Cecilia Milford, Kenneth Ngure, Sara Newmann, Nicholas B. Thuo, Susana Berrios, Mags E. Beksinska, Nelly Mugo, Larry Rand

**Affiliations:** 1 Department of Obstetrics, Gynecology and Reproductive Sciences, University of California San Francisco, San Francisco, CA USA; 2 MRU (MatCH Research Unit), Department of Obstetrics and Gynecology, Faculty of Health Sciences, University of the Witwatersrand, Durban, South Africa; 3 Department of Community Health, Jomo Kenyatta University of Agriculture and Technology, Nairobi, Kenya; 4 Center for Clinical Research, Kenya Medical Research Institute (KEMRI), Nairobi, Kenya; 5 University of Illinois College of Medicine, Chicago, IL USA

**Keywords:** Preterm labor, Preterm birth, Maternal health, Sub-Saharan Africa, Medical device development

## Abstract

**Background:**

Prematurity and its complications are the leading cause of death and disability in children under five in Africa and North America, affecting as many as one in ten pregnancies. Screening tests to predict preterm birth (PTB) are insensitive, costly, and often unavailable in low resource settings. In parallel with early-stage U.S.-based testing of a novel self-placed intravaginal device to predict PTB risk, we elicited key stakeholder input from two sub-Saharan African countries to ensure local contextual factors inform future development of the device and its acceptability.

**Methods:**

A qualitative study was conducted in Kiambu County, Kenya and KwaZulu-Natal Province, South Africa. We conducted 26 focus group discussions with pregnant women (n = 132) and males from the community (n = 54); in-depth interviews with women who had a history of PTB (n = 10), healthcare providers (n = 16), and health system experts (n = 10). Interviews were transcribed and thematic analysis was performed using an iterative coding technique. In addition, we facilitated user-centered design sessions to generate prototype preferences.

**Results:**

Women with a personal history of PTB were almost unanimous in support of the proposed device, whereas those with no experience of PTB expressed the greatest degree of reservation. Healthcare providers anticipated that women with a history of PTB would accept the device. However, various potential challenges were identified, including potential discomfort with device insertion, hygiene, and sexual activity, as well as need for provider training, and attention to country-specific regulatory processes. Both community participants and providers expressed a preference for a provider inserted device. Design recommendations included preference for a small, soft, pliable device, with a shape that could facilitate easy removal.

**Conclusions:**

Use of an intravaginal device to detect risk of PTB was generally acceptable, however stakeholders expressed a notable preference for insertion by providers. This reflects the significance of end-user consultation in device design and use. Recommended device modifications as well as educational messaging and provider technical assistance may facilitate utilization.

## Plain language summary

Prematurity and its complications are the leading cause of death and disability in children under 5 years of age. Up to ten percent of pregnancies in North America and Africa are affected. Screening tests to predict preterm birth (PTB) are insensitive, costly, and often unavailable in low resource settings. A U.S.-based study has developed an intravaginal device to detect preterm labor. In order to assess acceptability and guide development for a low-resource setting, a qualitative study was conducted in Kenya and South Africa. We conducted focus group discussions and interviews with pregnant women, male community members, health care providers and health systems experts. Participants with a personal history of PTB were almost unanimous in support of the proposed device; women with no experience of PTB expressed the greatest degree of reservation. Various potential challenges were identified: discomfort, hygiene, and sexual activity, the need for provider training and attention to country-specific regulatory processes. Design recommendations included preference for a small, soft, pliable device, with a shape that could facilitate easy removal. Stakeholders expressed a strong preference for insertion by providers. These findings reflect the significance of end-user consultation in device design and use.

## Background

Preterm birth (PTB) is a global health epidemic with more than 15 million premature births and 1 million associated deaths worldwide annually [1, 2]. In addition to being the leading cause of mortality among children under five, those who survive are at significant increased risk for long-term morbidity and lifelong disability [3–5]. Despite decades of research, the incidence of PTB has remained relatively unchanged, complicating up to 10–11% of all pregnancies in North America and Africa [6]. Currently, preterm labor is diagnosed when a woman becomes symptomatic with uterine contractions. This represents a physiologic endpoint of a long cascade of clinically silent, essentially irreversible events. Understanding of the signals that lead to preterm labor is limited and as such, by the time contractions are apparent, the process is difficult to arrest or reverse. In resource-rich settings, ultrasound to detect shortening cervical length and the fetal fibronectin (fFN) immunoassay may be used as risk prediction tests. However, these methods have low sensitivity, are useful only in risk assessment, and require both specialized training and costly equipment [7, 8]. Current treatments offered to those identified as at risk for preterm labor may include cervical cerclage and vaginal progesterone [9–11], however these are considered preventative measures and there are currently no reliable therapies to arrest preterm labor once it has begun. Novel strategies are urgently needed to identify women at risk for PTB much earlier in the disease process to facilitate timely intervention and guide the development of new therapies.

A proof-of-concept device to identify women at high risk of going into preterm labor has been developed and has been tested in a clinical study for a high resource setting at the University of California, San Francisco. This novel intravaginal device measures microscopic changes in tissue density to identify in real time the structural collagen changes of a woman’s cervix during pregnancy but well before symptomatic labor begins [12]. The design allows for a self-inserted device to fit over the cervix (similar to a cervical cap) and leverages inexpensive, low-power Bluetooth (rather than cellular signal) to send an alert to a mobile device (belonging to either the user or the provider) to warn of the newly detected changes and the increased risk of preterm labor. The device is designed to be used after 20 weeks of gestation. While the proof-of-concept design has been used to take isolated measurements in a clinic setting, the final product may be kept in situ for days or even weeks, similar to a pessary used for pelvic organ prolapse. If successful, such an alert during this “silent phase” of early parturition may open a new window of opportunity for evaluation and intervention that could delay or prevent the preterm birth. This is a critical period as a delay of even 1 to 2 weeks can result in dramatic improvement in long term outcomes for the child. Such a device may be of particular use in sub-Saharan Africa, where the incidence of PTB remains high and access to diagnostic modalities and tertiary care are limited. In addition, early warnings of cervical changes may allow women more time to access care, which in turn could lead to improved pregnancy outcomes [12].

While other studies have investigated attitudes toward medical interventions requiring vaginal contact, such as the female condom, vaginal rings, anti-viral foams or gels, SILCS diaphragm and the pessary, as well as intravaginal patches and pessaries during pregnancy, to our knowledge, no other study to date has evaluated attitudes towards an intravaginal device to detect risk of preterm labor designed for use during pregnancy [13–17]. Vaginal pessaries are not used routinely as a treatment to prevent preterm birth, but studies are on-going to assess whether there is a benefit to prophylactic use in certain high risk groups [18]. The adoption of any novel treatment or device is dependent on understanding and acceptance at the level of the healthcare system, provider, and most especially, the client. It should not be assumed that the acceptability and feasibility of the existing form factor (and conditions for use) being tested in the US could be extended elsewhere with unique cultural beliefs and potential systems barriers. The current form factor is in the shape of a cervical cap with an attached introducer. During the initial study, the device was inserted by a clinician, the cap fitted over the cervix, measurements obtained, and the device removed immediately. The proof-of-concept clinical trial has now been completed and data analysis is in process. Next steps of the project include design optimization of a wireless device without an introducer that can be used by either a clinician or end-user and left in situ. As such, prior to the introduction of a preterm labor risk-detection device, and to ensure optimized implementation in sub-Saharan Africa, we felt it critical to elicit stakeholder input and recommendations for an acceptable and feasible device.

We conducted an exploratory, qualitative study on attitudes and preferences regarding an intravaginal diagnostic device, focusing on women with a previous history of PTB, healthcare providers, health systems experts, and other men and women in the local communities with pregnancy experience. We also used processes inspired by the human-centered design method, which focuses on the engagement of key stakeholders and centering of end users’ needs, desires and context to define the ideal form and use of a novel device [19, 20].

## Methods

### Study setting

This study was conducted across two settings: Kiambu County in Kenya and KwaZulu-Natal province in South Africa, in late 2016 and early 2017. In both countries, health systems experts were recruited nationally, and healthcare provider and community level participants were recruited from sites in urban and peri-urban/rural areas. In South Africa, participants from the urban area were largely recruited from a large township bordering a coastal city centre. The peri-urban/rural location was closely located to a clinical trial site that was researching the efficacy of the Dapivirine vaginal ring [15]. In Kenya, participants from urban areas were recruited from an industrial town within which the trial site was located, 40 km northeast of the capital city. Peri-urban and rural participants were drawn from outlying settlements (which included informal settlements) and agricultural zones within Kiambu County.

### Data collection

Five target population groups were recruited independently via purposive or snowball sampling: Women with a history of PTB were recruited from healthcare facilities, via community health workers or word-of-mouth, either face-to-face or telephonically for in-depth interviews (IDIs). Adult and minor pregnant women receiving prenatal care in urban and rural/peri-urban healthcare facilities were selected according to pregnancy status (> 20 weeks gestation) and age (16–45 years). Where possible, women with a history of PTB were recruited. They were invited to participate in focus group discussions (FGDs), face-to-face at the healthcare facilities. Adult men (who had at least one child or a pregnant partner) were recruited through community male and youth groups and community health workers. Additional participants were male partners of participating community females. Where possible, men who had a partner with a history of PTB were recruited, they were not necessarily partners of women who had participated in the IDIs or FGDs. Some community males were recruited face-to-face, others via telephonic contact, for FGDs. Healthcare providers were recruited face-to-face from urban and rural/peri-urban healthcare facilities, and included a range in levels, including operational managers, doctors, nurses and community health workers, all with experience in maternity and/or antenatal services. They were invited to participate in IDIs. Healthcare providers and health systems experts were purposively selected based on knowledge and expertise with family planning and products such as medical devices, diaphragms, vaginal rings, intra-uterine devices (IUDs) and female condoms. This was to ensure that responses could be targeted and based on experience specific to reproductive health and use of products that are inserted vaginally. More specifically, health systems experts needed to be able to provide input on policies specific to the introduction and approval of medical devices. The health systems experts were recruited telephonically or via email, and included policy makers, health economics and regulatory stakeholders with expertise in family planning, and were invited to participate in IDIs.

Data were collected via IDIs with women with a history of PTB (5 to 8 interviews per country), healthcare providers (8 to 10 interviews per country, half rural/peri-urban, half urban), and health systems experts (5 to 8 interviews per country); and via FGDs with community women and men (with 6 to 11 participants per group). Community men and women FGDs were stratified by sex, age and by urban or rural/peri-urban location. FGDs are typically a relatively homogenous group of people comprised of 6 to 8 people, to generate information through discussions on specific topics [21]. IDIs and FGDs were conducted in English or local language (isiZulu in South Africa, and Kiswahili in Kenya), and were audio recorded with permission. Interviews and discussions were approximately 1–1.5 h in duration, and were conducted using semi-structured guides, which had exploratory questions with probes.

The interview guides for community members and women with a history of PTB included questions on general health, perceptions of PTB, the introduction of a device to detect PTB, use of a vaginal device, technological and access considerations, and device storage. In order to elicit responses about a vaginal device, participants were shown images of a pregnant women’s anatomy, with and without a device on the cervix, as well as images depicting a possible interaction between the vaginal device, a cell phone and healthcare providers. Following this they were asked to describe their thoughts on such a device, and these thoughts were then probed in more detail. The interview guide for health care providers included questions on general health and PTB, delivering antenatal care to women at risk for PTB, and questions about an intravaginal device—introduction, technological aspects and storage. They were also asked to describe their thoughts on such a device. Health systems experts were asked about policy and regulatory issues for the introduction of a device, as well as about cost and affordability, and distribution and access to such devices.

All participants were shown examples of two intra-vaginal devices, the FemCAP (cervical cap) and a pessary, for their information. These devices were used to demonstrate devices that are placed on the cervix and are approved for intra-vaginal use, therefore providing a useful visual reference point for the discussions about what a device for monitoring changes in the cervix should look like. A prototype SMART device was also shown to participants, but no details of the study being conducted in the USA were shared with participants [12, 22]. The community males and females were invited to describe and draw images of what they would perceive to be an ideal vaginally inserted PTB detection device [23, 24].

Research assistants of same language, gender and similar age (where possible) conducted FGDs and IDIs with community men and women and women with a history of PTB. Senior researchers and project coordinators conducted IDIs with healthcare providers and health systems experts. All had experience in conducting interviews and FGDs in the local communities, on sensitive topics related to sexual and reproductive health.

### Ethical and regulatory considerations

All participants provided voluntary, written informed consent, and where participants were under 18 years of age, parental consent and individual assent were obtained. Ethical approval was obtained from the Kenya Medical Research Institute (KEMRI) in Kenya (KEMRI/SERU/CCR/0028/3240), and the Human Research Ethics Committee (HREC) of the University of Witwatersrand in South Africa (M160131). In Kenya, additional support and approval was obtained from the Kiambu County Health Research and Development Unit and participating healthcare facilities. In South Africa, additional support and approval was obtained from the Department of Health (Provincial and District) as well as from participating healthcare facilities. Approval was also granted by the University of California San Francisco Institutional Review Board.

### Data analysis

IDIs and FGDs were transcribed and translated (where necessary) into English. The transcriber simultaneously translated and transcribed the local language audio—i.e., listened to the local language audio and translated and transcribed directly into English. Translation was word for word but allowed for revision of sentence construction and order to make grammatical sense. The translation and transcriptions were reviewed by another local language (usually the person who conducted the interview/discussion) expert and senior researcher.

A qualitative data analysis software program, Dedoose [25], facilitated organization and coding of data. Thematic analysis was used to explore attitudes towards an intravaginal PTB detection device as well as for recommendations regarding an ideal device. Codes were generated iteratively based on input from the questions in the guides as well as from emergent themes. Themes were identified by reading the transcripts and identifying repetitions, categorizations, metaphors, similarities and differences, and from theoretical interpretations of the data [26]. In addition, drawings were thematically coded to determine perceptions of an ideal PTB detection device [23].

Data analysis was conducted as a team effort across Kenya and South Africa, to enable the development of a code list that was applicable, reliable, and valid across the two country sites, which could allow cross-country comparisons of study findings [27]. A single draft code list was collaboratively developed across the two countries by reviewing transcripts from various target groups from both countries. The code list was tested via double coding across countries, and once there was agreement on code names and definitions, a master code list was finalized. Each country had two coders (led by CM and NBT respectively) and coded their own country transcripts (a portion (> 18%) of which were also double coded at a country level). There was ongoing communication about coding throughout data analysis, and any coding queries were collaboratively resolved. This process facilitated reliability and validity of coding between and within the two countries.

In the results, illustrative quotes from either country will be provided when there is agreement among both countries according to the thematic area discussed; and where there are inter-country differences, quotes from both countries will be included. Perspectives from different stakeholders will be presented in an integrated manner and factors associated with device acceptability as well as potential barriers will be identified. Where there are differences between stakeholder opinions, and within stakeholder groups (e.g. young versus older participants, urban versus peri-urban/rural) these will be highlighted.

## Results

There were 195 community member participants in total, of whom 104 were located in Kenya and the remaining 91 in South Africa. In depth interviews with five female participants with a history of PTB were conducted in each country; the remaining 3 per country participated in the focus group discussions. There were 9 males in South Africa, and 1 male in Kenya, who participated in the FGDs, who reported that they had a partner who had a history of PTB. The information from the IDIs with women with a history of PTB demonstrated sampling to redundancy, as no new information was described.

There were 142 female and 53 male community member participants (who participated in FGDs—8 female groups and 4 male groups in South Africa; and 12 female and 2 male groups in Kenya). Demographics between the two countries were similar with regard to age, parity, urban or rural location (Table 1). A greater proportion of Kenyan community member participants were employed and living with a partner in comparison to South Africa. The majority of Kenyan community member participants reported primary school as their highest education level as compared to South African community member participants, who were more likely to have completed secondary school. In both countries, the majority of community members were within 5 km of a healthcare facility and the most common mode of transportation was by foot with some variation between countries. The vast majority owned their own cellphone.

**Table 1:** Demographics of participants

	Kenya		South Africa	
	Female n (%)	Male n (%)	Female n (%)	Male n (%)
Community participants	87	17	55	36
Focus groups	82 (94)	17 (100)	50 (91)	36 (100)
In depth interviews	5 (6)	N/A	5 (9)	N/A
Age (range)	23 (17–40)	31 (24–40)	23 (16–37)	30 (21–42)
No. of children (range)	1 (0–5)	2 (0–5)	1 (0–3)	2 (0–7)
History of PTB	8 (9)	1 (6)	8 (15)	9 (25)
Urban	40 (46)	10 (59)	22 (40)	17 (47)
Rural	42 (48)	8 (47)	28 (51)	19 (53)
Employed	20 (23)	17 (100)	8 (15)	4 (11)
Education level				
Primary school	53 (61)	7 (41)	14 (25)	17 (47)
Secondary school	19 (22)	7 (41)	40 (73)	18 (50)
College/University	3 (3)	2 (3)	1 (2)	0 (0)
Relationship status				
Partner—cohabiting	62 (71)	15 (88)	11 (20)	9 (25)
Partner—not cohabiting	23 (26)	2 (12)	44 (80)	25 (69)
Proximity to healthcare centre				
≤ 5 km distance	72 (83)	16 (94)	40 (73)	34 (94)
> 5 km distance	16 (18)	1 (6)	15 (27)	2 (6)
Travel by foot	36 (41)	11 (65)	30 (55)	29 (81)
Travel by public transport	43 (49)	6 (35)	23 (42)	7 (19)
Has own cellphone:	67 (77)	16 (94)	51 (93)	35 (97)
Healthcare providers	8		8	
Provider type				
Managerial	0		1	
Physician	3		1	
Nurse	4		4	
Counselor/community health worker	1		2	
Female	5		7	
Mean age (range)	41 (29–55)		44 (27–61)	
Mean years in practice (range)	13 (2–26)		7 (0–20)	
Health systems experts	5		5	
Female	2		3	
Mean age (range)	42 (31–53)		52 (40–66)	
Mean years in current position (range)	6 (0–9)		6 (1–19)	

A total of eight healthcare providers in each country were recruited for participation. These healthcare providers were selected from facilities within which the community members were based. Provider types included those in managerial positions, physicians, nurses, and counselors or community health workers. In both countries, half of the providers were nurses, with 13 years average experience in Kenya and seven years in South Africa. The average age of the providers was similar between the two countries (41 years in Kenya and 44 years in South Africa) and the majority of respondents were women (five in Kenya and seven in South Africa). There were five health systems experts interviewed in each country; in total, half were women, and the average work tenure was 6 years.

Qualitative results are presented according to thematic areas which emerged from the data and address the aims of the manuscript, to describe overall perspectives on the ideal form and use of a device, including (1) factors promoting acceptability of an intravaginal device; (2) specific challenges with the introduction and use of a device; and (3) design preferences.

### Factors promoting acceptability of an intravaginal device

Of note, throughout the manuscript the use of the term “acceptability” refers to willingness or openness to the device either with regard to information or use.

#### Previous experience with preterm birth

Women who had previously experienced PTB were essentially unanimous in favor of a device that could detect a high-risk of going into preterm labor or that could potentially give them other information about the health of the fetus.

*“What would make me want to use it is that I would like it to tell me if there is [a] problem appearing.” (Female participant with history of PTB, South Africa)*

Other female respondents were generally positive but expressed more reservations than their counterparts with a prior history of PTB about both self-insertion as well as the concept of an intravaginal device. Concerns included insertion practice, hygiene and comfort, as described below. Healthcare providers also stated that they anticipated acceptance of such a device among women with prior PTB experience:

*“[E]specially for mothers who’ve had...previous preterm deliveries. It will be so helpful because that way they are able to know when they can run to the facility.” (Healthcare provider, Kenya).*

#### Male partner support

The majority of male partners interviewed responded positively to the concept of an intravaginal device. Partners stated that they were supportive of a device that could potentially be beneficial to the mother and baby:

*“I think I would like her to use it [intravaginal preterm birth detection device]; it’s best for her to insert it at a certain stage so that it can protect her…. To avoid problems of the baby being born before time.” (Male community member, South Africa).*

Some male partners expressed some concerns for the baby regarding safety and hygiene if using an intravaginal device.

#### Experience with intravaginal devices

Community members, providers, and health systems experts pointed to prior use of intravaginal devices as evidence for the acceptability of an intravaginal device to detect risk of PTB. Although not specifically asked about previous experience with intravaginal devices, participants spontaneously described these experiences. Pregnant participants who had previously used either the female condom, the menstrual cup, an intrauterine device (IUD), or the Dapivirine ring stated that they would be comfortable using another type of intravaginal device even during pregnancy. Health systems experts reported that prior success with the female condom suggested that with an adequate educational campaign, an intravaginal device for use during pregnancy could be successful:

*“[M]any of the things we have done that use the vagina have been a challenge, but they have been used. Like now female condoms is an issue, […] but they are still used, …so we need to give them cultural advice and tell them exactly what their benefits are.” (Health systems expert, South Africa).*

#### Perceived usefulness

Potential users, healthcare providers, and health systems experts at all sites expressed openness to an intravaginal device, as long as it had been proven to be of benefit and provide useful information about potential risk of PTB. Pregnant women without a history of PTB stated that they would be interested in using such a device as long as safety and efficacy had been demonstrated. Pregnant women also pointed to a timely alert as a potential benefit.

*“Maybe it tells you that something is happening in your womb you have to go to the clinic and check, than getting things late… it is going to be the one that encourages you to go and get help.” (Pregnant community member, South Africa).*

Similarly, healthcare providers stated that if the device could accurately alert patients to cervical changes it could encourage them to seek care early and potentially improve preterm birth outcomes.

*“I would say that if this technology will come in, it will be of great importance to our clients…they don’t need to worry even at home. They know when the problem will…in case of any problem it will be signaled to their phone then they can be…they can come to the hospital immediately for intervention.” (Healthcare provider, Kenya).*

### Specific challenges identified

#### Discomfort with vaginal touching and device insertion

Some interviewees expressed reluctance about device acceptability due to discomfort with vaginal touching and device insertion. This hesitation although cited by some female participants, was more frequently cited by providers and male community members. Respondents noted that different cultural beliefs could discourage vaginal touching and pointed to poor uptake of the female condom and low acceptance of vaginal examinations for Pap smears as evidence for potential reluctance among patients.

*“You see again in African setup you know if there’s anything going inside [intravaginally]… that system is not very welcome.[…], but having said that when you look at like we have not been able to market very well to uh, the, the, the female condom to prevent HIV infection.” (Health systems expert, South Africa)*

*“The concern that I see it’s very invasive, [….] So, they don’t like coming to the clinic and we are doing them PVs [pelvic examinations]. So, they know that no, this time I have to do the PV and so on and so on; it’s very invasive for them unless, unless it is a different woman, it’s a woman who have experienced this pre-term labor and can do anything to prevent it. Those women maybe, those women who won’t mind taking it off and on.” (Healthcare provider, Kenya).*

#### Device positioning, comfort, hygiene, and impact on sexual activity

Participants in both IDIs as well as FGDs, identified several potential challenges to the insertion of an intravaginal device. Potential users voiced concerns regarding pain or discomfort associated with either insertion or wearing of the device. For example:

*“What can scare you is the first time is that how is it going to be inserted and how is it going to be removed? I think that is what can be scary, that pain.” (Pregnant community member, South Africa).*

Concerns over harming the cervix or disrupting the pregnancy were also expressed.

*“Because there are some body parts you should not touch and you can touch them during insertion and cause harm or hurt the cervix.” (Pregnant community member without preterm birth experience, Kenya).*

The question of hygiene regarding device storage and cleaning was also raised. Some participants suggested that the device be left in place to avoid potential challenges of unhygienic conditions.

*“That is a place [referring to vagina] that requires high levels of hygiene. It [device] is better just inserted and left there because constant insertion and removal and disturbing the cervix might hurt her and she gets other side effects.” (Male partner, Kenya).*

Female participants also expressed concern regarding the impact the device might have on sexual activity. Some stated that they worried the device might be felt by their partner and that this might contribute to domestic discord.

*“If you cannot feel it when you are having sex with my partner it can stay inside there is no problem, but if he is going to feel it and he is going to ask me a lot of things, […] that I will not be able to respond to them and end up having a fight.” (Pregnant community member, Kenya)*

#### Need for buy-in, training, and support among providers

In addition to some of the technical considerations mentioned above, both providers and health systems experts cited potential challenges to the implementation of a preterm birth diagnostic device in health facilities. Health systems experts in both Kenya and South Africa pointed to the need for buy-in on the part of providers ranging from physicians, to nurses, to community health workers, in order to facilitate device introduction, use and success. The need for training was also articulated:

*“And nurses must go… must have in-service training just to assess if they’re still updated or if there are new developments or some new equipment. But what is extremely important is to have the equipment, have the spares and know where these things need to be repaired.” (Health systems expert, South Africa).*

Stakeholders also pointed to the challenge of providing on-going technical support to providers in their individual clinical settings.

#### Cell phones and Bluetooth/wireless technology

Respondents were concerned about the reliability of cell phones as a device to receive messages, as cell phones break or get lost, and network unreliability may cause delays in messages being received. Concerns were also raised that not all people have access to smart/android phones.

Healthcare providers had concerns about safety of Bluetooth technology and community members’ questions related to lack of understanding of how Bluetooth works.

*“[A]nd because we don’t know the side effect and using the wireless technology, so it’s still risky to that foetus, you don’t know in the long-term how it might affect that foetus, who might be born at term.” (Healthcare provider, South Africa).*

#### Country-specific health systems and regulatory processes

Multiple health systems and regulatory challenges were identified by health systems experts in both Kenya and South Africa. Due to the fact that the regulatory environment is continually changing, these challenges would need to be explored in more detail if/when device implementation was considered.

### Design preferences

#### Preference for provider-inserted device

Participants were asked their preferences regarding whom they thought would be best to insert an intravaginal PTB risk detection device, when given the choices of self-insertion, placement by healthcare providers, or by a trained non-healthcare provider such as a male partner. All healthcare providers in both locations agreed that a health professional would be the best person to insert the device, citing hygienic reasons and knowledge of vaginal anatomy. Similarly, women with a history of PTB preferred a healthcare professional due to concern for discomfort.

*“To me, I see that it’s a doctor or person who is trained to insert that device. […] No, I wouldn’t like to self-insert. […] Because the time I insert it and maybe if I feel the pain and I will then stop […] and end up not inserting it.” (Female participant with history of PTB, South Africa).*

Among focus group participants, the majority of both males and females preferred a healthcare professional to insert the device, and reasons cited were concerns over incorrect placement or the possibility of triggering preterm labor. Participants also felt that if a professional were to place the device, this would present an opportunity for increased monitoring.

*“If I visit the doctor and they use a device like this to check and they can advise me about my problem so the device is not bad; I think it is good.” (Pregnant community member, Kenya).*

Very few respondents in either location reported a preference for self-insertion and only a couple preferred a male partner to insert the device. Respondents cited lack of anatomical knowledge among non-healthcare professionals as well as the physical difficulty of self-insertion among pregnant women as barriers to insertion.

*“Because there are some body parts you should not touch and you can touch them during insertion and cause harm or hurt the cervix.” (Pregnant community member, Kenya)*

*“I think it must be health professionals, which can be people from the clinic or the doctor because they are the people trained to work with the inside of the person. If I were to say she must insert it herself maybe it will not be fixed to the position.” (Male community member, South Africa)*

#### Material, size, color, and shape preferences

The majority of respondents from both Kenya and South Africa felt that a soft, pliable device made from material similar to the FemCap (a reusable barrier contraceptive made of flexible silicone fitted over the cervix) and would promote ease and comfort for the user. A few respondents from both countries recommended that a range of sizes be available.

*“[My] suggestion is, if it is made, we are not the same, we don’t wear same underwear, so as women, if the sizes say in certain age people usually wear the size so on and so on, it must not be one size.*” *(Male respondent, South Africa)*.

Preference for device color was discussed—in both Kenya and South Africa, color was important, as healthcare providers felt it should be visible during physical exams.

*“It should have its own unique color. […] I think like it can even be green, blue so that at least if… it will be removed …by the time the pregnancy is at term, you are able …to see fast then come with it.” (Healthcare provider, Kenya).*

**Fig. 1:**
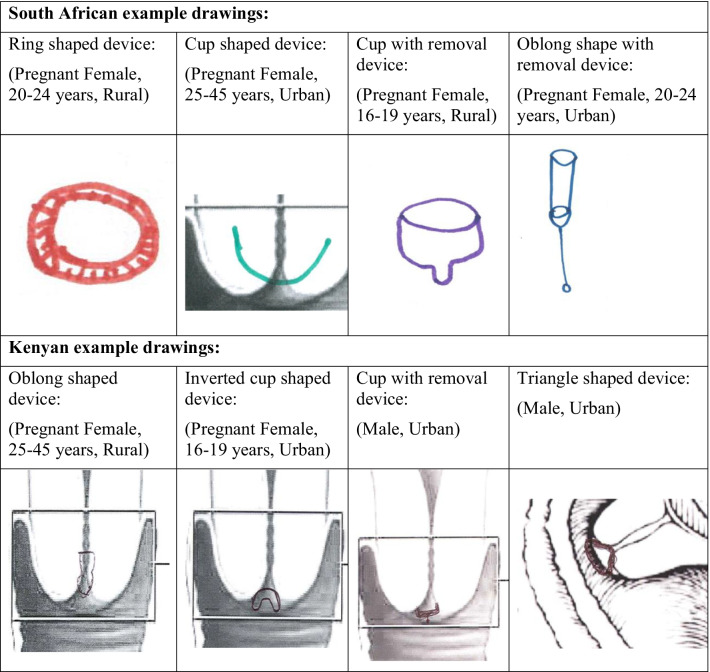
Examples of design preferences

Various device shapes were described and drawn—including a ring, cup, triangle, and oblong (see Fig. 1). Only South African respondents described that the device design should take into consideration factors to facilitate ease of insertion and removal—some community males and females suggested that it could be pen- or “thermometer”-shaped, and some women suggested a string/handle to facilitate removal:

*“I mean, it is better if it is a pen like, you see, so that it can be able to be inserted like this and maybe there must be something that is string like, so that it is easy that when you have inserted it you hold this string and take it out” (Pregnant community member, South Africa).*

## Discussion

There is general acceptability for the need and usefulness of an intravaginal PTB risk detection device across multiple stakeholder groups in both South Africa and Kenya. Our data demonstrate that women with a personal history of PTB were the most likely to be open to the proposed device. The majority of male partners also indicated openness to a device or indicated interest in further information. The group with the greatest hesitation was women without previous exposure to PTB. Healthcare providers were generally accepting of the proposed device, identified multiple potential benefits, and pointed to previous success with other intravaginal devices and a predictor of clinical uptake. Providers and health systems experts however, also identified specific challenges such as need for technical support and clinical training, as well as evolving country-specific regulatory processes.

A number of potential challenges were identified from all groups surveyed. Although there are clearly multiple challenges to overcome, many of these obstacles are likely able to be modified with appropriate community sensitization and education, provider training, and regulatory exploration.

The main concerns about using an intravaginal PTB detection device were related to the physical properties of such a device, and the impact it may have on sex, which can largely be addressed through device design as well as information dissemination, education, and community sensitization. These concerns about device use have been mirrored in a study about the SILCS Diaphragm in South Africa, in which it was also highlighted that education and information dissemination could address concerns about the physical properties of the device [24, 25]. Studies on vaginal rings have also demonstrated the importance of comfort and partner acceptability with use, and especially the impact on sex [28, 29]. Additionally, previous studies suggest that women have regularly been underestimated in their ability to care for and wear cervical barrier devices such as diaphragms appropriately [30, 31]. An intravaginal PTB detection device has the potential to change diagnostic procedures and impact outcomes of high-risk pregnancies, which has social and economic benefits, reducing the cost of PTB to both individuals and broader economies.

Importantly, participants expressed a clear preference for a provider-inserted device. Citing concerns related to hygiene and device storage, as well as expert training (such as fear of harming the woman or her pregnancy via misplacement), both women and male partners re-iterated this preference throughout the study. Given the reported acceptability of wearing such a device on a proposed long-term basis, this finding suggests that future design efforts should be focused on a “smart pessary” or pessary-inspired device that would be fitted and placed by a provider and worn for days or weeks at a time. Device design suggestions varied across individuals and groups and did not appear to be influenced by the example device designs (FemCAP and pessary), although there was a strong preference for a soft, pliable device, with a color that is visible during physical exams.

Besides contributing to the research and development of our proposed device, this study demonstrates unequivocally that participant-driven design processes are possible in low-resource settings [32]. Although efforts have been made to focus on the user experience for contraceptives and sexually transmitted infection prevention strategies, to our knowledge there has been no study yet that has examined end-user preferences for an intravaginal device that would be worn during pregnancy. We demonstrate here that working with potential users, their male partners, and health system stakeholders yields great insight into design preference that could significantly impact successful implementation and uptake, and that this process is clearly possible despite operating in a resource-poor environment.

As with any qualitative investigation, the findings presented here are limited in their generalizability. The attitudes and perspectives represented are localized to two distinct countries and are limited by the small sample size necessary for exploratory research. It should be noted that while many similarities were identified among the participants, caution should be used when extrapolating the specifics of these findings to other areas of the African continent or even to other communities outside of those directly surveyed within Kenya and South Africa. Yet, understanding local context and culture are important for end-user acceptability. To note, the majority of participants were community members, and although the community perspective is important for understanding adherence, the number of healthcare professionals interviewed were limited in comparison. Among health systems experts, no one in either country who was directly involved with the approval or licensure process for new medical devices, was available for consultation. Prior to rolling out a novel device in either country, more information on this process is needed.

## Conclusion

Proof of concept studies and future developments for this device are on-going, taking into consideration findings from this study. One of the biggest discoveries made was the strong preference for a provider-placed device, which is different from the originally envisioned use-case and serves as an excellent example of how stakeholder engagement can impact changes in design to ensure uptake of a culturally acceptable device. These findings demonstrate an openness to a proposed intravaginal preterm birth risk detection device among participants in two different sub-Saharan African contexts. Women with personal experience of PTB were the most accepting, followed by their male counterparts. Healthcare providers and women without prior PTB experience expressed the greatest degree of reservation. The most common concerns were focused around device safety, discomfort with device insertion, concern for hygiene, and impact on sexual activity. Needs for ongoing provider training and a greater understanding of country-specific regulatory processes were also highlighted. Results indicate that further design modification should reflect the preference for insertion by providers. Education for end-users and technical support for providers will be critical throughout clinical use of this device. These findings also underscore the importance of involving end-users and stakeholders in biomedical design and demonstrate the feasibility of doing so in a low-resource setting.

## Data Availability

The datasets used and/or analysed during the current study are available from the corresponding author on reasonable request.

## Abbreviations

HREC: Human Research Ethics Committee
fFN: Fetal fibronectin
FGD: Focus group discussion
IDI: In-depth interviews
IUD: Intrauterine device
KEMRI: Kenya Medical Research Institute
PTB: Preterm birth
PV: Pelvic examination
